# Patient-reported outcome measures and physical function following head and neck lymphedema — a systematic review

**DOI:** 10.1007/s11764-024-01683-3

**Published:** 2024-09-26

**Authors:** Katrina Gaitatzis, Belinda Thompson, Fiona Tisdall Blake, Louise Koelmeyer

**Affiliations:** Australian Lymphedema Education, Research & Treatment (ALERT) Program, Department of Health Sciences, Faculty of Medicine, Health and Human Sciences, Macquarie University, Sydney, NSW Australia

**Keywords:** Head and neck cancer, Lymphedema, Patient-reported outcomes, Physical assessments, Quality of life, Systematic review

## Abstract

**Purpose:**

Head and neck cancer (HNC) treatments often lead to significant morbidity, including lymphedema. This systematic review aims to comprehensively explore the prevalence and impact of head and neck lymphedema (HNL) following treatment.

**Methods:**

A systematic literature search was conducted up to September 2023. Studies evaluating HNL prevalence, associated factors, impact, patient-reported outcomes (PROMs), and physical assessments were included. Methodological quality assessment was performed, and data were synthesised narratively.

**Results:**

Twelve studies met the inclusion criteria, with methodological quality ranging from moderate to high. Internal lymphedema prevalence was consistently higher than external lymphedema, with varying rates attributed to treatment modalities and assessment methods. PROMs such as the Lymphedema Symptom Intensity and Distress-Head and Neck and physical assessments including Patterson’s Rating Scale were commonly utilised. HNL significantly impacted quality of life and physical function, with reported symptoms including discomfort, tightness, swallowing difficulties, and psychological distress.

**Conclusion:**

HNL is a common sequela of HNC treatment with significant implications for individuals’ QoL. Standardised assessment protocols and tailored interventions are needed to address the needs of individuals with HNL and improve overall outcomes.

**Implications for Cancer Survivors:**

This systematic review highlights a significant prevalence of lymphedema, particularly internal lymphedema in the larynx and pharynx, following treatment. Swallowing difficulties, nutritional issues, anxiety, depression, and body image concerns were associated with both internal and external lymphedema. The impact on quality of life is substantial, with survivors experiencing physical symptoms and psychosocial challenges, emphasising the importance of integrated care approaches tailored to both aspects of well-being.

**Supplementary Information:**

The online version contains supplementary material available at 10.1007/s11764-024-01683-3.

## Purpose

In 2022, 660,000 new cases of head and neck cancer (HNC) were reported annually, making it the seventh most prevalent cancer worldwide [1]. With a reported 350,000 deaths, HNC comprises approximately 1 to 2% of all tumour-related deaths [2]. Over 90% of HNC are squamous cell carcinomas, typically originating in the pharynx, larynx, or oral cavity [3]. While surgery or radiotherapy alone is effective for 60–95% of individuals diagnosed with early-stage disease, most individuals initially present with locally advanced disease (stage III or IV) [2]. For these individuals, aggressive multimodal treatments, usually consisting of surgery followed by either adjuvant radiation therapy or concurrent chemoradiation, are required and lead to a disease control rate at 5 years of approximately 40% [4]. While advances in surgical techniques, such as robotic surgery, have decreased tracheotomy procedures and hospital stay, mortality and morbidity, as well as late effects following treatment such as lymphedema remain high [3].

Lymphedema, resulting after HNC treatment, is characterised by an accumulation of lymphatic fluid in the extracellular space due to lymphatic system insufficiency and has been reported to affect between 12 and 90% of individuals [5–10]. Head and neck lymphedema (HNL) can occur both externally (face, submental region, and neck) and internally (oral cavity, larynx, and pharynx) and can impact breathing, face, neck, and shoulder movement, psychological function, body image, and lead to poor quality of life (QoL) [5]. Lymphedema, along with surgical interventions for HNC can contribute to disfigurement, which has been reported to be one of the leading causes of negative body image, often categorised as one’s self-observation along with other’s reactions [11]. Body disfigurement has been reported as high as 20% and is higher in individuals with HNL due to the disfigurement being highly visible [11, 12]. Individuals who experience disfigurement are often stigmatised impacting them socially and economically. This, combined with their reduced ability to eat or speak, can impact interpersonal relationships negatively [11, 12].

Lymphedema has been shown to advance from soft edema usually of the submental and/or neck region to fibrotic tissue when left untreated [8]. Post-radiation fibrosis has been identified as a contributor to the development of fibrotic tissue and has been associated with dysphagia and trismus, impacting QoL [8]. To date, there has been no consensus on which QoL or physical assessment measures are important for assessing individuals with HNL [13]. However, early surveillance models of care for those at risk of lymphedema, specifically in other cancer populations such as breast cancer, have been shown to have a positive clinical impact in minimising chronic lymphedema and potentially fibrotic development [5]. Therefore, understanding the trajectory of HNL and risk factors associated with the development is important in establishing consistent and reliable assessments for HNL for those most at risk [5, 8].

This systematic review aims to comprehensively explore the outcomes and implications of HNL following treatment for HNC. This review investigates the prevalence, associated factors, and impact of HNL, as well as exploring patient-reported outcomes (PROMs) and physical assessments.

## Methods

### Literature search

**Fig. 1:**
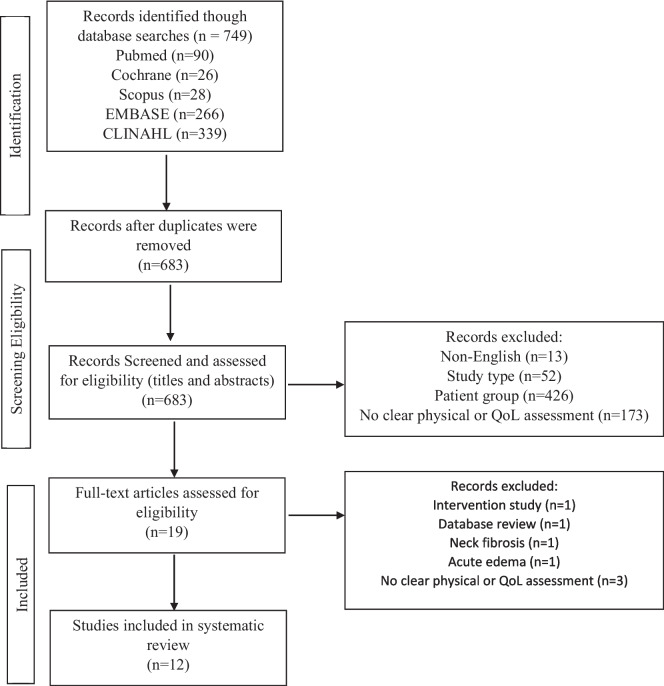
Flow chart of data extraction and synthesis

A literature search of six databases (Scopus, Embase, PubMed, Cochrane Central Register of Controlled Trials, and CINAHL) was completed up to 9th September 2023 to explore outcomes following HNL. The subject heading head and neck cancer was used in combination with surgery, radiotherapy, treatment, lymphedema, external lymphedema, internal lymphedema, body composition, muscle mass, fat mass, malnutrition, undernutrition, quality of life, LSIDS, PROMs and physical function (supplementary file 1). Studies were limited to full text, human and English, with systematic and literature reviews being excluded. Figure 1 depicts the study identification and selection process.

### Study inclusion

Individuals over 18 years of age, with a confirmed diagnosis of HNC, who had undergone treatment and experienced lymphedema in the head and neck region were included. Studies were included if PROMs and physical measurements for HNL were analysed. Studies were excluded if they did not include HNC.

### Data extraction and quality assessment

One reviewer (KG) conducted the electronic data searches, after which, two reviewers (KG, BT) independently evaluated titles and abstracts based on inclusion criteria. If reviewers could not reach a consensus on the discrepancies in study selection, a third reviewer (LK) was involved.

Studies included in the review were scored using the McMasters University Guidelines and Critical Review Form for Quantitative Studies, with a scoring system ranging from 0 to 15, where seven to nine is moderate quality, and 10 or more is indicative of high-quality studies [14]. Full-text articles were independently rated (KG and BT), 1 (yes) or 0 (no) for each category, with total scores tallied. For each study included: demographics, medical and treatment information, QoL and/or physical assessment method used, study inclusion/exclusion criteria, and study conclusions were documented.

## Results

### Study selection

This systematic search initially identified 749 articles. After removing duplicates, 683 titles and abstracts were reviewed based on the inclusion and exclusion criteria, 664 articles were removed, and 19 full texts were retrieved and assessed for eligibility. Of these, 12 were accepted for inclusion in this systematic review (Fig. 1).

### Methodological quality

The included articles were published between 2009 and 2023. Using the McMasters methodological quality assessment, studies included in this review were evaluated for quality and scores ranged from eight to twelve. High methodological quality, a score of ten or higher was shown for eight of the twelve studies, with the remaining four showing moderate quality (Table 1). None of the twelve studies addressed contamination or co-intervention, and only six justified sample size [15–20]. Only one out of the twelve studies were retrospective [16], with none being randomised studies. Seven studies investigated both PROMs and physical assessments for HNC [16–18, 20–23], three examined physical assessments only [10, 19, 24], and two examined PROMs only [15, 25].

**Table 1:** Critical review. The total possible scores for each category are shown in brackets

Author(s)	Study purpose (1)	Literature (1)	Design (1)	Sampling (2)	Outcomes (2)	Intervention (3)	Results (4)	Conclusions (1)	Score (/15)	Quality
Queija et al. (2020)	1	1	1	1	0	1	2	1	8	Moderate
Deng et al. (2021)	1	1	1	1	0	1	2	1	8	Moderate
Deng et al. (2013)	1	1	1	1	2	1	3	1	10	High
Deng, Ridner, Murphy et al. (2012)	1	1	1	2	0	1	3	1	10	High
Schiefke et al. (2009)	1	1	1	1	2	0	2	1	9	Moderate
Deng et al. (2016)	1	1	1	2	2	1	3	1	12	High
Deng et al. (2022)	1	1	1	1	2	1	2	1	10	High
Pigott et al. (2023)	1	1	1	2	2	1	1	1	10	High
Jeans et al. (2020)	1	1	1	2	2	0	2	1	10	High
Jeans et al. (2021)	1	1	1	2	1	1	3	1	11	High
Jeans et al. (2023)	1	1	1	2	1	1	3	1	12	High
Deng, Ridner, Dietrich et al. (2012)	1	1	1	1	1	1	2	1	9	Moderate

### Study characteristics

Summary of individuals demographics, measures used, and findings are summarised in Table 2. All included studies included individuals with HNC. A total of 958 individuals were included. Six studies [18–22, 25] reported a mean age range of 59 to 63.7 years, while six studies [10, 15–17, 23, 24] reported a median age range of 59 to 67 years, with total number of participants enrolled in each study ranging from 46 to 163. Eleven out of the twelve studies documented treatment regimens completed [10, 15–22, 24, 25]. All twelve studies documented tumour site and ten documented stage (I-IVb) [10, 15–17, 19–23, 25], with the other two studies documenting T, N, and M classification only [18, 24]. One study examined individuals with HNL [15]. Eleven of the included studies had individuals who completed surgery with/without chemoradiotherapy (CRT), post-operative radiotherapy (PORT), or radiotherapy alone, while one study did not mention treatment modalities [23].

**Table 2:** Summary of included studies investigating patient-reported measures and physical assessments

*Author(s)*	*Population*	*Study design*	*Mean/median age*	*Number of individuals*	*PRO’s*	*Physical assessments*	*Outcomes/findings*
Queija et al. (2020) Brazil	Squamous cell carcinoma of the upper aerodigestive tract, surgical treatment ≥3 months ago	Prospective cross-sectional	Median = 61, range, 29–82	*N* = 46	None	Circumferential measurement, FEES, Foldi’s stages of lymphedema scale, Patterson’s Radiotherapy Edema Rating Scale	97.8% of individuals had lymphedema, with the majority having combined internal and external (73.9%). The neck region had the greatest occurrence of lymphedema (71.7%). External lymphedema: individuals treated with radiotherapy had higher incidence of submandibular lymphedema. Internal lymphedema: treatment combined with radiotherapy had higher incidence of edema in almost all structures. Even minimal swelling compresses nearby structures affecting deglutition and speech.
Deng et al. (2021) USA	Tumour in oral cavity and oropharynx (≥stage II) Aged ≥21	Prospective, longitudinal	Mean age = 59 (SD = 10.6, range, 27–82)	*N* = 117	LSIDS-H&N	None	LSIDS demonstrated an ease of readability, with 82 individuals (70.1%) agreeing the survey captured their symptoms, however, 67 individuals stated the survey was burdensome to complete. 64-item survey was reduced to a 33-item survey (with 7 symptom clusters) after analysis. The end of treatment demonstrated the highest burden of symptoms with 3-12 months posttreatment showing reduced symptom domain scores. At 12-months post-treatment, no statistical significance was shown in symptom burden from baseline.
Deng et al. (2013) USA	3-months post head and neck cancer treatment, aged ≥18, free of active cancer	Prospective, correlational study	Mean age = 59.8, range, 33.1–86.7	*N* = 114	VHNSS HADS BIS FACT-H&N	FEES, Foldi’s stages of lymphedema scale, Patterson’s Radiotherapy Edema Rating Scale,Range of motion	On examination, 46.6 (*n* = 48) had external lymphedema, either stage 1 (*n* = 21) or stage 2 (*n* = 27), with the neck and submental areas being the most common sites Of 81 individuals with endoscopies, 19 had mild, 25 moderate, and 11 severe internal lymphedema. Increasing treatment modalities increased symptom burden including swallowing-related symptoms. Younger individuals had higher anxiety. BIS was associated with external (*p* = .024) not internal (*p* = .866) lymphedema severity. Time of diagnosis were inversely associated with forward flexion (*p* = .014), left lateral flexion (*p* = .015), left lateral rotation (*p* = .003), and right lateral rotation (*p* = .032). Time since treatment were inversely associated with forward flexion (*p* = .014), left lateral flexion (*p* = .028), left lateral rotation (*p* = .003), and right lateral rotation (*p* = .032). Neck rotation was statistically significantly correlated with severity of lymphedema. FWB and HNCS subscales of the FACT-H&N demonstrated the strongest specific multiple correlations of lymphedema severity.
Deng, Ridner, Murphy et al. (2012) USA	Phase 1: health care providers Phase 2: HNC patients, secondary lymphedema, aged ≥18, ≥3 months post-treatment completion, no evidence of disease	Prospective correlation study	Phase 1: median = 61.96, range,49.57–77.02 Phase 2: median = 60.38, range, 40.43–81.37	*N* = 48	LSIDS-H&N	None	Phase 1: six domains were developed to create a 55-item survey. Phase 2: consisted of 67-items, all answered with a yes or no, and if yes was selected individuals scored 0 (not at all) to 10 (severe) the intensity and distress of each item. Feasibility: time for completion took 6–20 min Acceptance: 96.7% of individuals completed all 67-items Readability: individuals identified 6-items with some concern. Most common symptoms reported: feeling uncomfortable in one’s head or neck, tightness, stiffness, problems swallowing solids, firmness, voice changes, feeling tired, something stuck in one’s throat, problems sleeping and hoarseness.
Schiefke et al. (2009) Germany	Surgically treated oral squamous cell carcinomas (pN0) between 2000 and 2005, no evidence of disease	Prospective cross-sectional pilot study	Median = 67, range, 46–85	*N* = 49	EORTC QLQ-C30 QlQ-HN35 HADS	Self-developed score for cervical scar parameters, modified Miller score, score of May, constant shoulder score	No significance in QoL scores between SNB or SND groups. General health-related QoL was lower in both groups compared to the general population. Individuals undergoing SNB had significantly fewer swallowing problems (*p* = .043) HADS yielded no significant difference between groups. Fear of disease progression was greater in SND versus SNB (*p* = .024). Postoperative mobility was greatest in SND versus SNB groups: tactile epicritic sensitivity (*p* < .0001) and protopathic (pain) sensitivity (*p* < .0001). SNB group had fewer shoulder problems.
Deng et al. (2016) USA	Head and neck cancer diagnosis, >3-months post-treatment, aged ≥21, no evidence of cancer	Retrospective cross-sectional	Median age = 60.3, range, 33–87	*N* = 163	LSIDS-H&N	Foldi’s stages of lymphedema scale, Patterson’s Radiotherapy Edema Rating Scale	Lymphedema individuals versus non-lymphedema individuals had increased symptom prevalence for altered symptom sensation, musculoskeletal symptoms, head and neck-specific functioning, and site-specific swelling (*p* < 0.05). Individuals with lymphedema had higher distress and intensity scores for numbness, tightness, heaviness, and warmth (*p* < 0.05).
Deng et al. (2022) USA	Newly diagnosed oral cavity and oropharynx tumours, stage II or greater, aged ≥21	Prospective longitudinal study	Mean = 59	*N* = 120	EORTC-QLQ-C30 NDI VHNSS HADS MC-C HN-LEF-SI SF-MPQ	Skin and soft tissue assessment, Modified Patterson Scale	HN-LEF- SI average and maximum soft tissue and neurologic toxicity scores were strongly correlated with NDI, SF-MPQ, HADS, and VHNSS, demonstrating convergent validity.
Pigott et al. (2023) Australia	Diagnosis of oral cavity, nasopharyngeal, oropharyngeal, laryngeal or hypopharyngeal cancers, aged ≥18, CTx and RTx	Prospective observational cohort study	Median age = 63, range, 58–67	*N* = 46	EQ-5D-5L, EORTC QLQ-HN43	ALOHA, TDC, MDACC rating scale, Patterson’s Radiotherapy Edema Rating Scale	TDC assessed external lymphedema increased from baseline (41.9%) to 12-weeks post-radiotherapy (50.4%). Compared to normative values no external lymphedema was present at baseline. Significant increases from baseline to 12-weeks post-radiotherapy were shown for ALOHA measurements. At 12-weeks post-radiotherapy all individuals had lymphedema at a minimum of 8 sites. QLQ-HN43 showed increased score averages from baseline to end of radiotherapy, with scores declining at study final assessment.
Jeans et al. (2020) Australia	Diagnosis of oral cavity, nasopharyngeal, oropharyngeal, laryngeal or hypopharyngeal cancers, aged ≥18, CTx and RTx, between 1 and 3 years posttreatment	Prospective cohort study	Mean = 61.4	*N* = 62	None	Patterson’s Radiotherapy Edema Rating Scale, MDACC Rating Scale	Majority of individuals had internal lymphedema only (61%), combined internal and external lymphedema (35%) and external lymphedema only (2%), with 32% receiving prior lymphedema therapy. Inter-rater reliability for Patterson’s Scale ratings was moderate across all sites. External lymphedema frequent sites were submental region (34%) and neck (16%). Statistical significance was shown for combined internal and external lymphedema with post-operative chemoradiotherapy (*p* < .001) compared to internal lymphedema alone with chemoradiotherapy.
Jeans et al. (2021) Australia	Diagnosis of oral, laryngeal, or hypopharyngeal cancer, radiotherapy or chemoradiotherapy	Prospective cross-sectional study	Mean = 63.7	*N* = 79	VHNSS	MDACC Rating Scale, Patterson’s Radiotherapy Edema Rating Scale, FEES, PAS, MASA-C, FOIS	Inter-rater reliability for internal lymphedema: -substantial (Kw = 0.72) with true vocal cords, epiglottis, and posterior pharyngeal wall (Kw = 1, 0.87, 0.86, respectively) -valleculae, false vocal cords, and anterior commissure (Kw = 0.48, 0.55, 0.57, respectively) had the lowest agreement. Majority had internal lymphedema (68%), with 29% having external and internal combined, and 1% having external only. External lymphedema was most prevalent in the submental region and neck. FEES assessment showed 80% scoring normal PAS scores. Dysphagia using MASA-C was identified in 67% individuals. Significant positive relationships between PAS scores with external lymphedema (*p* < .004) and internal lymphedema (*p* = .006) were seen. Oral tumours had lower FOIS scores Significantly negative relationship between VHNSS swallow solids scores and external lymphedema (*p* = .037) and internal lymphedema (*p* = .014) was seen
Jeans et al. (2023) Australia	New diagnosis of oral, nasopharngeal, oropharyngeal, laryngeal or hypopharyngeal cancer, CRT planning	Prospective longitudinal cohort study	Mean = 59.9	*N* = 33	VHNSS	MDACC Rating Scale, ALOHA, TDC, Patterson’s Radiotherapy Edema Rating Scale, MASA-C, FEES, PAS	Inter-rater reliability for Patterson Radiotherapy edema Rating Scale ratings -substantial (Kw = 0.83) highest agreement sites: True vocal folds, cricopharyngeal prominence, and posterior pharyngeal wall (Kw = 1, 0.95, 0.91, respectively). -moderate (Kw = 0.44) highest agreement sites: aryepiglottic folds, epiglottis, and cricopharyngeal prominence (Kw = 0.69, 0.68, 0.64, respectively). Internal and external combined lymphedema was greatest 3-months post treatment (71%), with 58% still present at 6 months. By 12 months, internal lymphedema was mainly present (90%). Significant MDACC score reduction between 6 and 12 months. The most frequently affected site was the submental region. ALOHA and TDC measures also demonstrated significant improvements between 6 and 12 months. Internal lymphedema was identified at 3 months (96%), 6 months (84%) and 12 months (65%). Relationship between dysfunctional PAS, FOIS, and MASA-C scores were significant (*p* < 0.05)
Deng, Ridner, Dietrich et al. (2012) USA	Aged ≥18, ≥3 months post cancer treatment, no evidence of disease	Prospective cohort study	Median = 59.67, range, 33.08–86.65	*N* = 81	None	Foldi’s stages of lymphedema scale, Patterson’s Radiotherapy Edema Rating Scale	61 individuals (67.9%) had lymphedema, 37 (45.7%) had external lymphedema typically in the neck and submental areas. 55 (67.9%) had internal lymphedema.

*FEES* fiber-optic endoscopic evaluation of swallowing, *LSIDS* Lymphedema Symptom Intensity and Distress Survey, *VHNSS* Vanderbilt Head and Neck Symptom Survey, *HADS* Hospital Anxiety and Depression Scale, *BIS* 10-item Body Image Scale, *FACT-H&N* Functional Assessment of Cancer Therapy – Head & Neck, *LSIDS-H&N* lymphedema symptom intensity and distress survey-head and neck, *EORTC QLQ-C30* European Organization for Research and Treatment of Cancer quality of life questionnaire, *QlQ-HN35* quality of life questionnaire-head and neck, *ASIA* American Spinal Injury Association, *MC-C* Marlowe-Crowne Social Desirability Scale-Short Form C, *SF-MPQ* short-form McGill pain questionnaire, *NDI* neck disability index, *HN-LEF-SI* head and neck lymphedema and fibrosis symptom inventory, *ALOHA* assessment of lymphedema of the head and neck, *MDACC* MD Anderson Cancer Center, *PAS* Penetration-Aspiration Scale; *MASA-C*-Assessment of Swallowing Ability-Cancer, *FOIS* Functional Oral Intake Scale, *TDC* tissue dielectric constant

The majority of studies used one of the following PROMs: Lymphedema Symptom Intensity and Distress Survey for Head and Neck (LSIDS-H&N) [15, 16, 25], Vanderbilt Head and Neck Symptom Survey (VHNSS) [18, 20–22], Hospital Anxiety and Depression Scale (HADS) [21–23], and/or EORTC QLQ-C30 [17, 21, 23]. For physical measurements, the majority used Patterson’s Radiotherapy Edema Rating [10, 16–22, 24], MD Anderson Cancer Centre Lymphedema Rating Scale (MDACC) [17–20], and Foldi’s stages of lymphedema [10, 16, 22, 24].

Narrative form rather than qualitative analysis was deemed more suitable for the presentation of results due to the large variability of study design, measurement methods, and outcomes.

### Lymphedema prevalence

Of the 12 studies included in this review, nine examined both internal and external HNL [10, 16–22, 24]. However, one of these studies reported overall lymphedema incidence of 78.5%, without further distinguishing between internal and external lymphedema [16]. In another study [21], while they used the Modified Patterson Scale, internal lymphedema incidence was not reported as study participants were part of a further study. Therefore, these two studies were not included in the internal lymphedema incidence described below. Among the seven studies that provide lymphedema percentages, internal lymphedema prevalence was found to be highest in six studies [10, 17–20, 22].

Deng et al. (2013) found a 67.9% internal lymphedema prevalence (23.5% mild, 30.9% moderate, and 13.6% severe), with 46.6% external and 38.3% combined lymphedema prevalence. Most individuals had stage IVa (53.7%), squamous cell carcinoma (93.2%), with oropharyngeal tumours (47.3%) [22]. Treatment modalities were highest for surgery with CRT (29.1) and chemo-induction with CRT (29.1%), with lower rates for radiotherapy alone (1.9%) or surgery with radiotherapy (9.7%) [22]. Similarly, Jeans et al. (2020) reported a 61% internal and 35% combined lymphedema prevalence, but only a 2% external lymphedema prevalence. More individuals underwent CRT (74%) for oropharyngeal tumours compared to PORT (26%) with oral tumours [19], compared with the Deng et al. (2013) study. Individuals were mainly diagnosed with stage I-II (35%) or stage III-IVc (26%) [19].

Jeans et al. (2023) also found a higher internal lymphedema prevalence (29%) at 3-months, with 0% external lymphedema prevalence among individuals who completed CRT. The majority had stage IVa tumours (83%) that were HPV-positive (76%), with a 71% combined lymphedema prevalence at 3-months post-treatment, increasing to 90% internal lymphedema only at 12-months post-treatment [20]. In a further study, Deng, Ridner, Murphy, et al. (2012) similarly reported a higher internal lymphedema prevalence (67.8%) compared to external (45.7%). The majority of individuals completed treatment with surgery and CRT (32.1%) or chemo-induction with CRT (25.9%), with lower rates for surgery alone (9.9%) or radiotherapy alone (2.5%) [10]. Like Jeans et al. (2021), the majority of individuals were diagnosed as having stage IVa (60.5%) tumours originating in the oropharynx (42.5%) [18].

A higher prevalence of internal lymphedema (68%) was observed compared to external (1%) or combined (29%) lymphedema in individuals with oropharyngeal tumours primary treated with CRT (90%) laryngeal with radiotherapy (64%), and oral tumours with PORT (100%) [18]. Most tumours were HPV-positive for CRT (80%) compared to HPV-negative for radiotherapy (71%) and PORT (93%) [18]. Neck dissection, either unilateral (57%) or bilateral (21%), was only performed in individuals treated with PORT [18]. In contrast, another study found lower prevalence rates of 13% internal, 10.9% external, and a 73.5% combined lymphedema [24]. Individuals in this study had primary tumours in the mouth (28.3%) and oropharynx (23.5%), and a higher proportion underwent radiotherapy (63%) [24]. Most individuals also underwent neck dissection, primarily supraomohyoid (28.3%) without reconstructive surgery (73.9%) [24].

In contrast to the above findings, Pigott et al. (2023) found that all individuals had internal lymphedema in at least one site at 12-weeks PORT, with the highest moderate to severe internal lymphedema ratings at 6- and 12-weeks post-radiotherapy. However, visualisation of all sites was not possible, and there was a low inter-rater reliability for the valleculae and pharyngoepiglottic folds. Individuals mostly had stage I (30%) or stage II (30%) tumours [17]. All individuals completed radiotherapy, with 84% also receiving chemotherapy. Internal lymphedema was more prevalent than external lymphedema at 12-weeks post-radiotherapy (50.4% external lymphedema incidence) [17].

Internal lymphedema was associated with arytenoids (73.9%) compared to external lymphedema in the submandibular (63%) and neck regions (71.1%) [24]. Internal lymphedema was most frequently identified in the arytenoids (92%), epiglottis (84%), pharyngoepiglottic folds (76%), and aryepiglottic folds (76%) [18], with the epiglottis and pharyngoepiglottic folds often reported as severe (18% and 14%, respectively). HNL was present in at least eight of the 13 internal sites for 56% of the individuals [18].

For external lymphedema, most individuals had MD Anderson Cancer Center (MDACC) staging of stage 0 (28.3%) or 2 (23.9%) for neck lymphedema, and stage 1 (37%) or stage 3 (26.1%) for submandibular lymphedema [24]. Using Foldi’s ‘Stages of Lymphedema’, individuals with external lymphedema in the Deng et al. (2013) study were classified as stage 1 (20.4%) or stage 2 (26.2%). Jeans et al. (2018) also found external HNL most common in the submental and neck regions (28% and 11%, respectively).

### Time since diagnosis

The analysis of critical time points for the development of lymphedema in three studies [10, 16, 22] revealed varying durations post lymphedema diagnosis and completion of HNC treatment. Two of these studies [10, 22] reported mean onset times of 2.58 months and 2.55 months, ranging from 0.5 to 14.6 years and 0.45 to 14.58 years, respectively. The mean duration since completion of HNC treatment was 27.4 months and 24.71 months, with a range of 3.1–156.4 to 3.09–156.39 months, respectively. Furthermore, matching individuals in lymphedema and non-lymphedema groups in the Deng et al. (2016) study revealed a median follow-up duration from HNC treatment of 12.6 months (range, 2-87) in the lymphedema group and 17.1 months (range, 3-77) in the non-lymphedema group.

### Methods for QoL assessment for HNL

Lymphedema Symptom Intensity and Distress-Head and Neck (LSIDS-H&N)

Nine studies evaluated PROMs [15–17, 19–23, 25]. One study developed and tested a self-report Lymphedema Symptom Intensity and Distress-Head and Neck (LSIDS-H&N) tool to address the need for a validated assessment tool for lymphedema [15]. This two-phase study used both health care providers (phase 1) and individuals with HNC (phase 2), resulting in a 67-item survey with four subscales. Individuals reported discomfort, tightness, stiffness, and psychological distress, with 50% experiencing fatigue despite being post-treatment (median of 19.66 months post HNC treatment) [15]. Feasibility, acceptance, and satisfactory readability were found [15]. The majority of individuals (70.1%) found the survey captured their symptoms and could help provide information to their health care providers; however, 60% also found the survey length to be burdensome [15]. Refinement led to a 33-item Head and Neck Lymphedema and Fibrosis Symptom Inventory (HN-LEF), with good reliability and content validity [25]. Differences in symptoms were noted between individuals with and without lymphedema, with those with lymphedema experiencing higher intensity and/or distress burden for numbness (*p* = 0.032), heaviness (*p* = 0.018), warmth (*p* = 0.036), pain (*p* = 0.036), swallowing (*p* = 0.019), trouble breathing (*p* = 0.009), and blurred vision (*p* = 0.018) [16]. A difference of 15% prevalence for the intensity and/or distress of symptoms between individuals with or without lymphedema was shown [16]. These differences remained statistically significant even after controlling for confounding variables such as age, tumour location, stage, and duration since HNC treatment completion [16].

### Head and Neck Lymphedema and Fibrosis Symptom Inventory (HN-LEF-SI)

Deng et al. (2022) examined the correlation of the Head and Neck Lymphedema and Fibrosis Symptom Inventory (HN-LEF-SI) with other validated assessments in individuals with advanced stage IV (42.8%) oropharynx tumours (61.5%) who were also HPV-positive (81%) [21]. The HN-LEF-SI average and maximum scores for soft tissue and neurological toxicity subscales showed convergent validity with the Neck Disability Index (NDI), Vanderbilt Head and Neck Symptom Survey (VHNSS), Hospital Anxiety and Distress Scale (HADS), Short-Form McGill Pain Questionnaire (SF-MPQ), and general pain and neck range of motion scores [21]. Inverse correlations were observed between HN-LEF-SI and the European Organization for Research and Treatment of Cancer Quality-of-Life Questionnaire (EORTC-QLQ-C30) [21]. Additionally, positive correlations were found between HN-LEF-SI systemic symptoms and social function (mood disorders, fatigue, and general function) and were also observed with NDI, SF-MPQ, HADS, EORTC-QLQ, as were body image and sexuality scores [21].

### Vanderbilt Head and Neck Symptom Survey (VHNSS)

The strongest correlation was reported between HN-LEF-SI swallowing and taste change scores with VHNSS symptom scores, indicating convergent and divergent validity of HN-LEF-SI [21]. The VHNSS, which assesses self-perceived symptom burden in swallowing and nutrition, showed relationships between lymphedema severity and swallowing difficulties, nutrition problems, voice, and dry-mouth-related symptoms. Symptom burden is characterised as either mild (scores 1–3), moderate (scores 4–6), or severe (scores 7–10) [20].

One study found the VHNSS subscale scores ranging from 0.00 to 3.00 indicated individuals with mild symptom burden who were in the recovery stages following HNC treatment [22]. Despite this, lymphedema severity and increased swallowing difficulties, nutrition problems, voice, and dry-mouth-related symptoms were all statistically significant [22]. Swallowing and poor nutrition were associated with external lymphedema severity (*p* = .006, *p* = .006) rather than internal lymphedema (*p* = .216, *p* = .516, respectively) [22]. Whereas internal lymphedema severity was associated with voice self-reported problems (*p* = .014) more than external lymphedema (*p* = .946) [22]. However, another study found a significant negative relationship between VHNSS swallowing scores for both external lymphedema (*p* = .037), as well as internal lymphedema severity (*p* = .014), demonstrating it could be the severity of lymphedema that increases the patient-reported symptom burden level, rather than lymphedema location (i.e. internal or external) [18]. For dry-mouth problems, both internal (*p* = .014) and external (*p* = .074) lymphedema were significantly related [22].

Jeans et al. (2023) found a significant relationship between external lymphedema and VHNSS swallow solids subscales, internal HNL severity score, and total internal HNL sites (*p* < 0.05). However, this same study found a higher association between HNL variables and other assessments (e.g. PAS, FOIS, and MASA-C scores) [20]. Whereas Deng et al. (2013) found a statistically significant relationship between increased number of HNC treatment modalities with increased swallowing-related symptoms (*p* = .012) and mouth-related symptoms (*p* = .003). An inverse relationship was also found between age and self-reported pain (*p* = .004) [22]. QLQ-HN43 showed an increase in self-reported symptom burden (‘dry mouth and sticky saliva’) at the start of radiotherapy (baseline), with scores returning to baseline levels at 12-weeks post-radiotherapy [17]. Lymphedema distress while lowest at 6-weeks post-radiotherapy increased at 12-weeks post-radiotherapy [17].

### Anxiety, depression, body image, and fear of progression

The Body Image Scale (BIS) demonstrated significantly higher body image disturbances for both single or widowed individuals compared to those who were married (*p* < .001), as well as those living in urban versus rural areas (*p* = .046) [22]. Younger individuals tended to report higher levels of anxiety, demonstrating an inverse relationship between age and anxiety (*p* = .001) [22]. This was further supported by the Functional Assessment Cancer Therapy-Head & Neck (FACT-H&N) demonstrating a statistically significant relationship between lower social well-being for single or widowed individuals (*p* = .003), and higher physical (*p* = .003), emotional (*p* = .001), and functional well-being (*p* = .027) with increased age [22]. Interestingly, one study found that the statistically significant association observed in individuals with HNC and anxiety, depression, and body image, were attributed to external (*p* = .024), not internal lymphedema (*p* = .866) [22].

No significant differences were found in anxiety and depression between individuals with sentinel node biopsy (SNB) removal of the first draining lymph node(s), and sentinel node dissection (SND) which included removal of lymph nodes in levels I–III [23]. No differences in overall or subscale scores (pain, sense, speech, and sexuality) for EORTC-QLQ-C30 were found between SNB and SND; however, individuals with SNB reported fewer issues swallowing (*p* = .043) [23]. A correlation between HADS and fear of progression was shown, with individuals with SNB scoring higher than individuals with SND; however, with increasing age, the fear of progression decreased [21]. Pigott et al. (2023) also reported fear of progression decreased at 12-weeks post-radiotherapy towards pre-radiotherapy levels.

### Neck mobility and function

Using the Cervical Range of Motion device to assess the degree of neck movement, the length of time following HNC diagnosis was associated with reduced neck flexion (*p* = .014), left lateral flexion (*p* = .015), left lateral rotation (*p* = .003), and right lateral rotation (*p* = .032) [22]. Time post HNC treatment was also associated with reduced neck flexion (*p* = .014 for both), left lateral flexion (*p* = .028), left lateral rotation (*p* = .003), and right lateral rotation (*p* = .032) [22]. Lymphedema severity was also statistically significant in relation to neck rotation [22].

Reduced neck mobility was more significant in individuals who had undergone neck dissection (76.2%) versus no neck dissection (26.87%) (*p* = 0.027) [24]. This was further supported by Schiefke et al. (2009), who found fewer functional shoulder issues, with symptom score, active shoulder function scores, and postoperative function being better for individuals who underwent SNB compared to SND.

## Discussion

This systematic review aimed to consolidate the existing literature on PROMs and physical assessment methods following HNC treatment. By critically analysing twelve relevant studies, this review provides insights into the prevalence, assessment methods, and impacts of HNL on individuals QoL and physical function.

The findings of this review highlight the substantial prevalence of HNL among individuals undergoing treatment for HNC. For internal lymphedema, Deng et al. (2016) stated that internal lymphedema measured through the fiber-optic endoscopic examination (FEES) and graded using the Patterson’s Radiotherapy Edema rating scale, is the only measure able to assess edema in the larynx and pharynx. Moderate inter-rater reliability and good intra-rater reliability were recorded for three studies [16, 19, 22]. Internal lymphedema was consistently reported to be more prevalent than external lymphedema, with varying rates across studies. Interestingly, one study which recruited individuals prior to radiotherapy and followed them up for 12 weeks, compared to most studies which recruited at least 3-months post HNC treatment completion had a much higher incidence of all individuals with internal lymphedema. However, it is also important to note, that while all individuals completed radiotherapy, a huge proportion also completed chemotherapy in this study; therefore, it cannot be distinguished which treatment modality led to the higher incidence of internal lymphedema.

Differences in prevalence rates reported in the literature may be attributed to differences in tumour types, treatment modalities, and assessment methods used across studies. Despite this, there is an increase in internal versus external lymphedema prevalence across all studies included in this review which distinguished between internal and external lymphedema [10, 17–20, 22]. Six studies supported a higher internal lymphedema prevalence with CRT for HNC [10, 17–20, 22]. Nevertheless, the prevalence rates emphasise the clinical significance of HNL and the necessity for effective assessment and management strategies.

Regarding assessment methods, the review highlights the lack of consensus on standardised measures for diagnosing and staging HNL. While several studies utilised PROMs such as the LSIDS-H&N and VHNSS, physical assessments varied widely among studies. Commonly employed physical assessment tools included FEES, Patterson’s Rating Scale, and Foldi’s stages of lymphedema [10, 16–22, 24]. However, the lack of uniformity in assessment tools poses challenges in comparing findings across studies and establishing a standardised approach to HNL assessment.

The impact of HNL on QoL and physical function emerged as a significant theme in the reviewed literature. Individuals with HNL reported a range of symptoms including discomfort, tightness, stiffness, pain, swallowing difficulties, and psychological distress. Interestingly, assessing self-reported swelling decreased from ‘a little’ at pre-radiotherapy to ‘not at all’ by 6-weeks post-radiotherapy in one study [17]. A possible explanation for these results could be recovery following completion of radiotherapy. These symptoms not only contribute to physical impairment but also have profound psychosocial implications, including body image disturbance, anxiety, depression, and fear of disease progression. The association between HNL severity and decreased neck mobility further highlights the multifaceted impact of HNL on individuals’ overall well-being.

Despite the valuable insights provided by the included studies, several limitations warrant consideration. Firstly, the heterogeneity in study designs, individual characteristics, and assessment methods limits the generalizability of findings. Additionally, most studies included in this review had moderate to high methodological quality; however, the absence of randomised controlled trials and the limited consideration of confounding variables in some studies may introduce bias. Furthermore, the lack of longitudinal studies impedes our understanding of the long-term trajectory of HNL and its impact on individuals’ outcomes over time.

Moving forward, future research in this area should aim to address the identified gaps by adopting standardised assessment protocols, employing longitudinal study designs, and investigating the efficacy of interventions aimed at mitigating the burden of HNL. Collaborative efforts between researchers, clinicians, and individuals with lived experience of HNL are essential to advance our understanding of this complex condition and improve outcomes for affected individuals. Moreover, the development of tailored interventions targeting both physical and psychosocial aspects of HNL is warranted to optimise individual care and enhance the overall QoL for this population.

## Supplementary Information

Below is the link to the electronic supplementary material.

Supplementary file1 (DOCX 17.3 KB)

Supplementary file2 (DOCX 16.5 KB)

## Data Availability

No datasets were generated or analysed during the current study.
