# Correction: Patient-reported outcome measures and physical function following head and neck lymphedema — a systematic review

**DOI:** 10.1007/s11764-025-01831-3

**Published:** 2025-05-16

**Authors:** Katrina Gaitatzis, Belinda Thompson, Fiona Tisdall Blake, Louise Koelmeyer

**Affiliations:** Australian Lymphedema Education, Research & Treatment (ALERT) Program, Department of Health Sciences, Faculty of Medicine, Health and Human Sciences, Macquarie University, Sydney, NSW Australia

**Correction: J Cancer Surviv**

10.1007/s11764-024-01683-3

In the Results section of this article in the sub heading Lymphedema prevalence the sentence “Pigott et al. (2023) also found a higher internal lymphedema prevalence (68%) with only 1% external and 29% combined lymphedema prevalence among individuals who completed CRT (64.5%), radiotherapy (17.7%), and PORT (17.7%)” should have read “Jeans et al. (2023) also found a higher internal lymphedema prevalence (29%) at 3-months, with 0% external lymphedema prevalence among individuals who completed CRT”

In addition in the sentence beginning “The majority had stage IVa tumours (83%) that were HPV-positive…” The reference “[17]” cited instead of “[20]”. The correct sentence should read as “The majority had stage IVa tumours (83%) that were HPV-positive (76%), with a 71% combined lymphedema prevalence at 3-months post-treatment, increasing to 90% internal lymphedema only at 12-months post-treatment [20]”.

In the sentence beginning “Like Pigott et al. (2023), the majority of individuals were diagnosed….” the reference “Pigott et al. (2023)” cited instead of “Jeans et al. (2021)” and the reference “[17]” cited instead of “[18]”. The correct sentence should read as “Like Jeans et al. (2021), the majority of individuals were diagnosed as having stage IVa (60.5%) tumours originating in the oropharynx (42.5%) [18].”

The original online version of this article was revised.

