# Supplementary material for: Patient-reported outcome measures and physical function following head and neck lymphedema — a systematic review
Source: J Cancer Surviv. 2024 Sep 26;20(2):738–51. doi: 10.1007/s11764-024-01683-3 (PMC12989013; doi:10.1007/s11764-024-01683-3)
Supplement: Supplementary file 1 — Supplementary file1 (DOCX 17.3 KB) [file 11764_2024_1683_MOESM1_ESM.docx]

**Journal of Cancer Survivorship**

**Patient-reported outcome measures and physical function following head and neck lymphedema – a systematic review**

¹Katrina Gaitatzis, ¹Belinda Thompson, ¹Fiona Tisdall Blake, ¹Louise Koelmeyer.

^1^ Australian Lymphoedema Education, Research & Treatment (ALERT) Program, Department of Health Sciences, Faculty of Medicine, Health and Human Sciences, Macquarie University, Sydney, NSW, Australia

**Supplementary File 1:** Search Strategy:

| Database | Date | Search Strategy |
| --- | --- | --- |
| Scopus | Until 9^th^ September 2023 | 1. Head and neck cancer OR head OR neck 2. surgery OR radiotherapy OR chemotherapy OR treatment 3. lymphoedema OR swelling OR lymphedema OR external lymphoedema OR external lymphedema OR internal lymphoedema OR internal lymphedema OR lymphatic changes OR edema 4. body composition OR muscle mass OR fat mass OR malnutrition OR undernutrition OR quality of life OR LSIDS OR lymphedema symptom intensity and distress survey OR patient reported outcome measures OR patient reported outcomes measure OR patient-reported outcome measure OR physical function OR BIS OR bioimpedance spectroscopy OR bioimpedance analysis OR lymph scanner OR TDC OR moisture meter OR tissue analysis OR percent water content OR PWC OR symptom assessment OR clinician reported outcomes 5. filter 3: Humans, Full text, English |
| Embase | Until 9^th^ September 2023 | 1. Head and neck cancer OR head OR neck 2. surgery OR radiotherapy OR chemotherapy OR treatment 3. lymphoedema OR swelling OR lymphedema OR external lymphoedema OR external lymphedema OR internal lymphoedema OR internal lymphedema OR lymphatic changes OR edema 4. body composition OR muscle mass OR fat mass OR malnutrition OR undernutrition OR quality of life OR LSIDS OR lymphedema symptom intensity and distress survey OR patient reported outcome measures OR patient reported outcomes measure OR patient-reported outcome measure OR physical function OR BIS OR bioimpedance spectroscopy OR bioimpedance analysis OR lymph scanner OR TDC OR moisture meter OR tissue analysis OR percent water content OR PWC OR symptom assessment OR clinician reported outcomes 5. filter 3: Humans, Full text, English |
| PubMed | Until 9^th^ September 2023 | 1. Head and neck cancer OR head OR neck 2. surgery OR radiotherapy OR chemotherapy OR treatment 3. lymphoedema OR swelling OR lymphedema OR external lymphoedema OR external lymphedema OR internal lymphoedema OR internal lymphedema OR lymphatic changes OR edema 4. body composition OR muscle mass OR fat mass OR malnutrition OR undernutrition OR quality of life OR LSIDS OR lymphedema symptom intensity and distress survey OR patient reported outcome measures OR patient reported outcomes measure OR patient-reported outcome measure OR physical function OR BIS OR bioimpedance spectroscopy OR bioimpedance analysis OR lymph scanner OR TDC OR moisture meter OR tissue analysis OR percent water content OR PWC OR symptom assessment OR clinician reported outcomes 5. filter 3: Humans, Full text, English |
| Cochrane Central Register of Controlled Trials | Until 9^th^ September 2023 | 1. Head and neck cancer OR head OR neck 2. surgery OR radiotherapy OR chemotherapy OR treatment 3. lymphoedema OR swelling OR lymphedema OR external lymphoedema OR external lymphedema OR internal lymphoedema OR internal lymphedema OR lymphatic changes OR edema 4. body composition OR muscle mass OR fat mass OR malnutrition OR undernutrition OR quality of life OR LSIDS OR lymphedema symptom intensity and distress survey OR patient reported outcome measures OR patient reported outcomes measure OR patient-reported outcome measure OR physical function OR BIS OR bioimpedance spectroscopy OR bioimpedance analysis OR lymph scanner OR TDC OR moisture meter OR tissue analysis OR percent water content OR PWC OR symptom assessment OR clinician reported outcomes 5. filter 3: Humans, Full text, English |
| CINAHL | Until 9^th^ September 2023 | 1. Head and neck cancer OR head OR neck 2. surgery OR radiotherapy OR chemotherapy OR treatment 3. lymphoedema OR swelling OR lymphedema OR external lymphoedema OR external lymphedema OR internal lymphoedema OR internal lymphedema OR lymphatic changes OR edema 4. body composition OR muscle mass OR fat mass OR malnutrition OR undernutrition OR quality of life OR LSIDS OR lymphedema symptom intensity and distress survey OR patient reported outcome measures OR patient reported outcomes measure OR patient-reported outcome measure OR physical function OR BIS OR bioimpedance spectroscopy OR bioimpedance analysis OR lymph scanner OR TDC OR moisture meter OR tissue analysis OR percent water content OR PWC OR symptom assessment OR clinician reported outcomes 5. filter 3: Humans, Full text, English |
