# Supplementary material for: Patient-reported outcome measures and physical function following head and neck lymphedema — a systematic review
Source: J Cancer Surviv. 2024 Sep 26;20(2):738–51. doi: 10.1007/s11764-024-01683-3 (PMC12989013; doi:10.1007/s11764-024-01683-3)
Supplement: Supplementary file 2 — Supplementary file2 (DOCX 16.5 KB) [file 11764_2024_1683_MOESM2_ESM.docx]

**Journal of Cancer Survivorship**

**Patient-reported outcome measures and physical function following head and neck lymphedema – a systematic review**

**¹Katrina Gaitatzis, ¹Belinda Thompson, ¹Fiona Tisdall Blake, ¹Louise Koelmeyer.** ^1^

Australian Lymphoedema Education, Research & Treatment (ALERT) Program, Department of Health Sciences, Faculty of Medicine, Health and Human Sciences, Macquarie University, Sydney, NSW, Australia

**Supplementary file 2:** Articles excluded after full-text review

| Author(s) | Title | Grounds for Exclusion |
| --- | --- | --- |
| Turcotte, et al. 2018 | Analysis of pharyngeal edema post-chemoradiation for head and neck cancer: Impact on swallow function, 3(5), 377-383 | Acute edema only. |
| Saleem, et al. 2023 | Quantifying Neck Fibrosis: Establishing the Domain Structure of the Neck Fibrosis Scale, 133(9), 2198-2202 | Neck fibrosis |
| Stubblefield, et al. 2023 | Under recognition and treatment of lymphedema in head and neck cancer survivors - a database study, 31(4) | Database review of lymphedema incidence |
| Aulino, et al. 2018 | Evaluation of CT changes in the head and neck after cancer treatment: Development of a measurement tool, 16(1), 69-74 | No clear physical or quality of life assessment tools used |
| Deng, et al. 2015 | Development and preliminary testing of head and neck cancer related external lymphedema and fibrosis assessment criteria, 19(1), 75-80 | No clear physical or quality of life assessment tools used. |
| Deng, et al. 2021 | Photobiomodulation Therapy in Head and Neck Cancer-Related Lymphedema: A Pilot Feasibility Study, 20. | Intervention study |
| Deng, et al. 2016 | Perceived Symptom Experience in Head and Neck Cancer Patients with Lymphedema, 19(12), 1267-1274. | No clear physical or quality of life assessment tools used |
